# Altitude-dependent *Bartonella quintana* Genotype C in Head Lice, Ethiopia

**DOI:** 10.3201/eid1712.110453

**Published:** 2011-12

**Authors:** Emmanouil Angelakis, Georges Diatta, Alemseged Abdissa, Jean-François Trape, Oleg Mediannikov, Hervé Richet, Didier Raoult

**Affiliations:** Université de la Méditerranée, Marseille, France (E. Angelakis, O. Mediannikov, H. Richet, D. Raoult);; Institut de Recherche pour le Développement, Dakar, Sénégal (G. Diatta, J.-F. Trape);; Jimma University, Jimma, Ethiopia (A. Abdissa)

**Keywords:** Pediculus humanus humanus, P. h. capitis, Ethiopia, altitude-dependent, genotype C, Bartonella quintana, bacteria, vector-borne infections, zoonoses

## Abstract

To determine the presence of *Bartonella quintana* in head and body lice from persons in different locations in Ethiopia, we used molecular methods. *B. quintana* was found in 19 (7%) genotype C head lice and in 76 (18%) genotype A body lice. *B. quintana* in head lice was positively linked to altitude (p = 0.014).

Head (*Pediculus humanus capitis* de Geer) and body lice (*Pediculus humanus humanus* Linnaeus) have been parasites of humans for thousands of years (*1*). Genetic studies based on mitochondrial DNA (mtDNA) have found 3 phylotypes of *P. humanus* (*2**,**3*). Clade A is the most common genotype with worldwide distribution and is found among both head and body lice. Clade B comprises only head lice and has been found in South America, Europe, and Australia, whereas clade C comprises only head lice from Ethiopia and Nepal (*2**,**4**,**5*). Only body lice have been implicated as vectors of *Bartonella quintana,* which causes trench fever, bacillary angiomatosis, endocarditis, chronic bacteremia, and chronic lymphadenopathy (*6*). However, *B. quintana* has been identified in head lice from homeless children in Nepal (*7*), in head lice from homeless adults in San Francisco, California, USA (*8*), and recently in head lice nits from a homeless man in Marseille, France (*9*). The objective of our study was to use molecular methods to determine the presence of *B. quintana* infection in head and body lice collected from patients in different locations in Ethiopia, a country where epidemiologic and clinical studies of zoonoses are scarce and a widespread louse infestation exists (*6*). Moreover, we assessed whether a phylogenetic difference existed between head and body lice collected from the same patient.

## The Study

After obtaining ethical approval from Jimma University Ethics Review Board, we collected head and body lice from persons at locations at different altitudes in Ethiopia. Lice were transferred to Marseille in sterile tubes at room temperature. Each louse was rinsed twice in sterile water for 15 minutes, and then total genomic DNA was extracted from each louse by using a QIAamp Tissue kit (QIAGEN, Hilden, Germany) as described by the manufacturer. Samples were screened by using a quantitative real-time PCR that targeted a portion of the *Bartonella* 16S-23S intergenic spacer region (*10*) and a specific *B. quintana* gene, *fabF3*, encoding 3-oxoacyl-(acyl-carrier-protein) (*9*). Negative controls (DNA from noninfected lice and sterile water) and positive controls (DNA from *B*. *elizabethae*) were included in each assay. From 50 randomly selected persons, each of whom had at least 3 body lice and 3 head lice; we selected 1 head louse and 1 body louse from each person for amplification and sequencing of the mitochondrial gene cytochrome b as described (*11*). For phylogenetic analysis, we used MEGA3.1 software (www.megasoftware.net). For data comparison, we used Epi Info version 6.0 software (Centers for Disease Control and Prevention, Atlanta, GA, USA). A p value <0.05 was considered significant.

**Figure A1:**
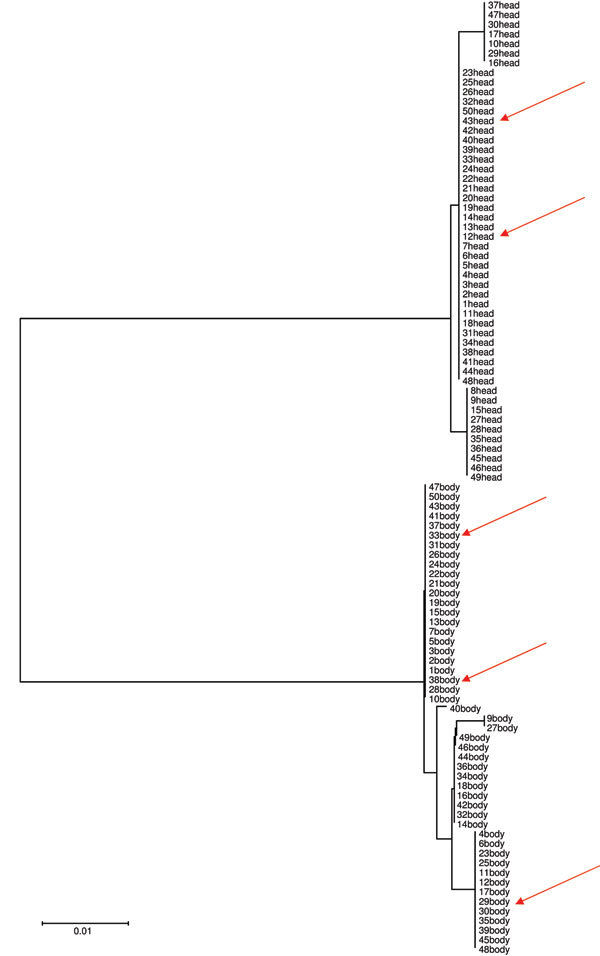
Neighbor-joining tree based on cytochrome b gene sequencing of head and body lice collected from 50 persons, Ethiopia. Arrows indicate lice positive for *Bartonella quintana*.

Overall, we tested 271 head and 424 body adult lice collected from 134 persons (109 women). Head and body lice co-infection existed on nearly all persons with a range of louse infestation of ≈5 to 20 lice per person. On 1 person, >100 lice were found. All but 2 persons had only black head lice; those 2 persons each had 1 gray louse among their populations of black head lice. All body lice were gray-transparent. All black head lice belonged to genotype C, and all gray lice belonged to genotype A (GenBank accession nos. JF694384–JF694442‏). Head and body lice grouped in 2 different clusters (Figure A1).

*B. quintana* was identified in 19 (7%) head lice collected from 9 (6.7%) patients and in 76 (18%) body lice collected from 17 (12.7%) patients (Table). Positive and negative controls showed expected results in all tests. No difference between male and female patients was observed in infestation with *B. quintana* (p = 0.36). No patients had both head and body lice infected with *B. quintana.* Significantly more body lice than head lice were infected with *B. quintana* (p<0.001). Significantly more body lice were infected with *B. quintana* in Dembi and in Tum than in the other locations (p = 0.001 and p = 0.02, respectively). *B. quintana* in body lice was not significantly linked to altitude (p = 0.3 with the Kruskal-Wallis test).

**Table:** *Bartonella quintana* in body and head lice from 7 different locations in Ethiopia

Location (altitude)	No. persons tested	No. head lice tested/no. persons	No. body lice tested/no. persons	No. (%) head lice with *B. quintana*	No. (%) body lice with *B. quintana*	No. (%) persons with head lice	No. (%) persons with body lice
Mizan Teferi (1,451 m)	35	66/29	53/32	0	0	0	0
Agaro (1,560 m)	15	19/15	25/15	0	2 (8)	0	1 (7)
Dembi (1,679 m)	19	34/15	187/19	0	55 (29)	0	11 (57)
Tum (1,830 m)	18	54/18	52/15	0	15 (28)	0	4 (26)
Balt (2,050 m)	12	19/12	44/12	0	4 (9)	0	1 (8)
Tikemit Eshet (2,121 m)	18	32/15	20/12	6 (18)	0	3 (20)	0
Gibarku (2,395 m)	17	47/17	43/17	13 (27)	0	6 (35)	0
Total	134	271/121	424/122	19 (7)	76 (18)	9 (7)	17 (14)

Only in the locations with the highest altitudes (Gibarku, altitude 2,395 m, and Tikemit Eshet, altitude 2,121 m) did we find *B. quintana* in head lice (Figure). At these higher altitudes, we did not find *B. quintana* in body lice. As a result, in Gibarku and in Tikemit Eshet, we found significantly more head lice that were infected with *B. quintana* than in the other locations (p = 0.0003 and p = 0.002, respectively). The presence of *B. quintana* in head lice was positively and significantly linked to altitude (p = 0.014 with the Kruskal-Wallis test).

## Conclusions

Our results confirm previous studies that show that genotype C head lice are prevalent in Ethiopia. Genotype C head lice cohabited with genotype A body lice, but we did not find an infestation of genotype A head lice in the persons studied. Only 2 persons each had 1 gray louse (genotype A) among many black head lice (genotype C). As a result, dual transmission cycles of lice appear to be occurring, and genotype C head lice may be inhibiting outbreaks of genotype A head lice.

We found that 7% of the head lice and 18% of the body lice from persons from Ethiopia were infected with *B*. *quintana*. For a long period, *B*. *quintana* was found only in body lice (*12*). It was first identified in head lice from homeless children in Nepal, where genotype C head lice exist (*4**,**7*). Additionally, *B. quintana* was recently found in lice from 138 homeless persons in the San Francisco area; 25% of head lice–infested persons had lice pools infected with *B*. *quintana* (*8*). However, we were unable to determine whether the study in Nepal reported *B*. *quintana* infection of genotype C or genotype A head lice (*7*) or whether the study in San Francisco reported infection of genotype A or genotype B head lice (*8*).

All persons with head lice infected with *B*. *quintana* were from locations with higher altitudes (>2,121 m), whereas at these altitudes, no body lice were infected with *B*. *quintana*. The permanent foci of body lice occur in regions subject to cold weather and in poverty-stricken areas such as Ethiopia. Body lice were found in 39% of Ethiopian migrants, and head lice were found in 65% (*13*), whereas 67% of schoolchildren from 3 different cities in Ethiopia harbored body lice (*14*). We do not have current hypotheses to explain the links from *B. quintana* to head lice at high altitudes or the mutual exclusion of *B. quintana* in head or body lice in the same person.

In conclusion, we identified *B. quintana* in genotype C head lice from Ethiopia only at altitudes >2,121 m and only in patients without infected body lice. However, further epidemiologic studies with head lice collected from more patients and from different countries should be performed to determine whether this altitude dependence of *B. quintana* in head lice exists only in Ethiopia or is a general phenomenon.

## Figures and Tables

**Figure:**
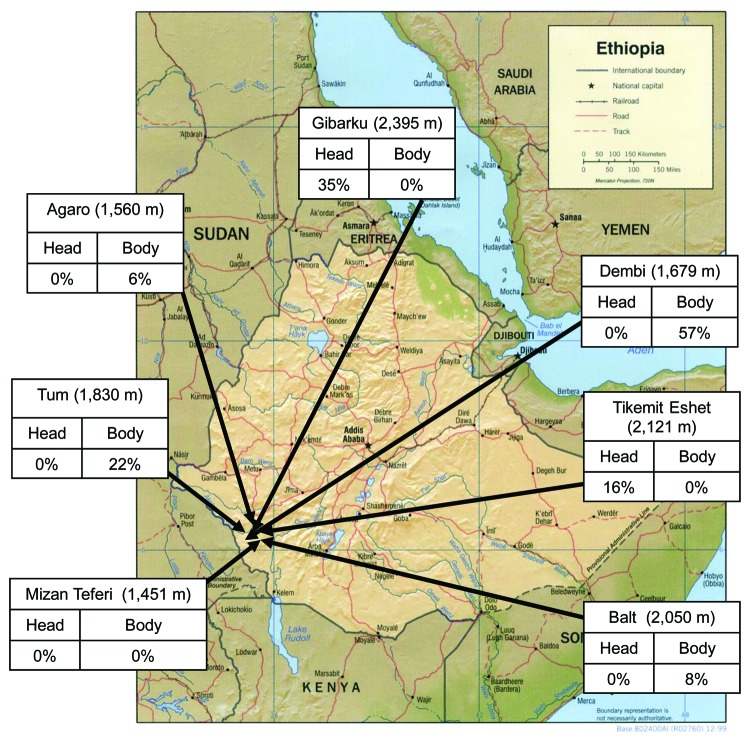
Percentage of humans with body or head lice infected with *Bartonella quintana* at different altitudes, Ethiopia.
